# Does functional MRI detect activation in white matter? A review of emerging evidence, issues, and future directions

**DOI:** 10.3389/fnins.2014.00239

**Published:** 2014-08-08

**Authors:** Jodie R. Gawryluk, Erin L. Mazerolle, Ryan C. N. D'Arcy

**Affiliations:** ^1^Division of Medical Sciences, Department of Psychology, University of VictoriaVictoria, BC, Canada; ^2^Department of Radiology, Faculty of Medicine, University of CalgaryCalgary, AB, Canada; ^3^Applied Sciences, Simon Fraser UniversityBurnaby, BC, Canada; ^4^Fraser Health Authority, Surrey Memorial HospitalSurrey, BC, Canada

**Keywords:** functional magnetic resonance imaging, white matter, brain connectivity, corpus callosum, internal capsule

## Abstract

Functional magnetic resonance imaging (fMRI) is a non-invasive technique that allows for visualization of activated brain regions. Until recently, fMRI studies have focused on gray matter. There are two main reasons white matter fMRI remains controversial: (1) the blood oxygen level dependent (BOLD) fMRI signal depends on cerebral blood flow and volume, which are lower in white matter than gray matter and (2) fMRI signal has been associated with post-synaptic potentials (mainly localized in gray matter) as opposed to action potentials (the primary type of neural activity in white matter). Despite these observations, there is no direct evidence against measuring fMRI activation in white matter and reports of fMRI activation in white matter continue to increase. The questions underlying white matter fMRI activation are important. White matter fMRI activation has the potential to greatly expand the breadth of brain connectivity research, as well as improve the assessment and diagnosis of white matter and connectivity disorders. The current review provides an overview of the motivation to investigate white matter fMRI activation, as well as the published evidence of this phenomenon. We speculate on possible neurophysiologic bases of white matter fMRI signals, and discuss potential explanations for why reports of white matter fMRI activation are relatively scarce. We end with a discussion of future basic and clinical research directions in the study of white matter fMRI.

## Motivation to investigate white matter fMRI

**Figure 1:**
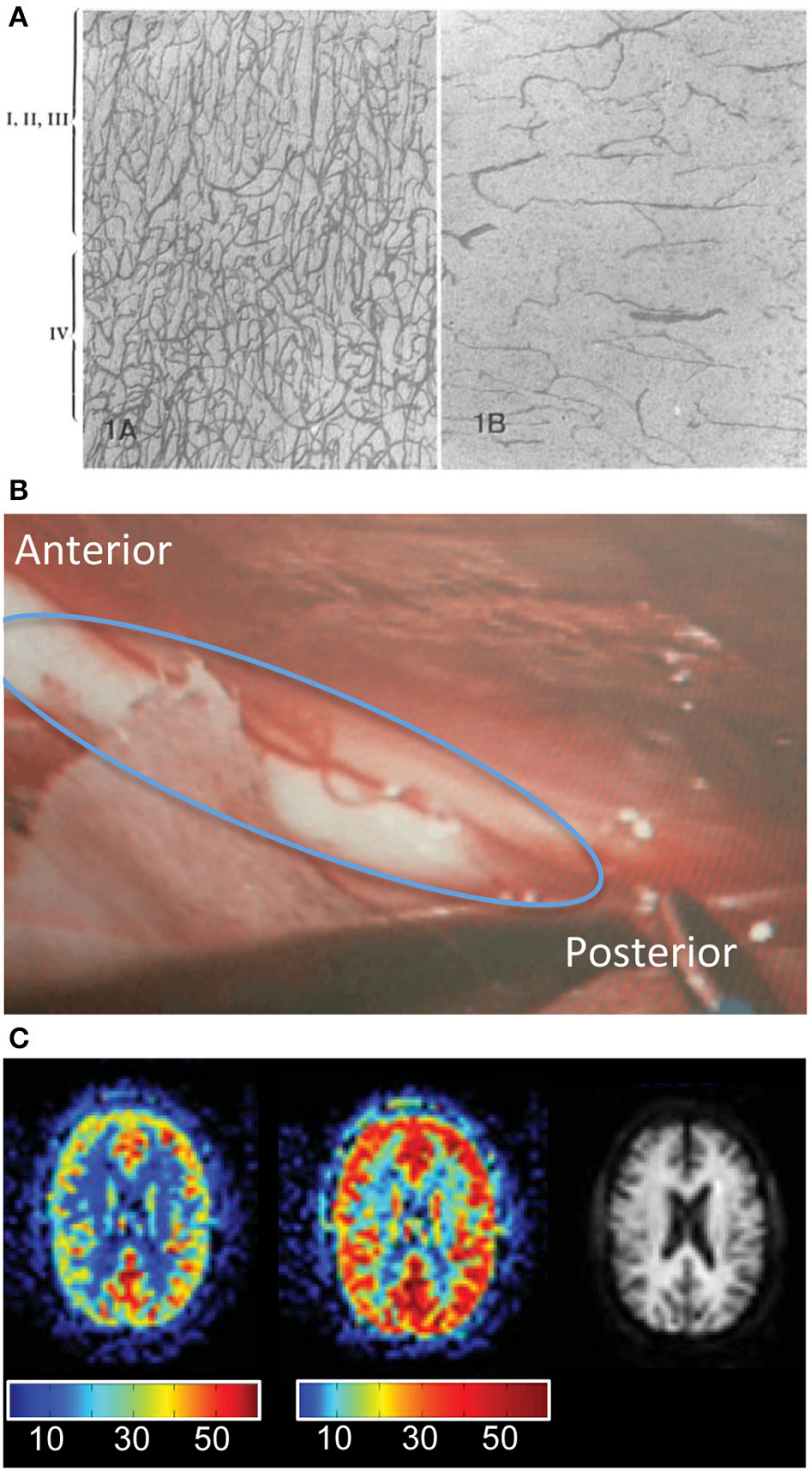
**The vasculature and perfusion of the white matter of the human brain**. **(A)** Capillary density for various layers of the cortex (left panel) compared to white matter (right panel). Fixed human brains were embedded in paraffin, sectioned (10 microns) and stained by means of Goldner's trichrome-method (reproduced with permission; Lierse and Horstmann, 1965). **(B)** Blood vessels on the surface of the corpus callosum (circled in blue). Photo captured during neurosurgery for corpus callosotomy (photo credit: R. D'Arcy). **(C)** Detection of white matter perfusion with arterial spin labeling MRI. Cerebral blood flow maps with different scale bars in order to better view gray matter perfusion (left) and white matter perfusion (middle). The right image shows the anatomy for reference (reproduced with permission; Van Osch et al., 2009).

Functional magnetic resonance imaging (fMRI) is used to visualize the neuroanatomical regions associated with brain function. The most commonly used technique for fMRI, blood oxygenation level dependent (BOLD) contrast, was first demonstrated in the early 1990s (Ogawa et al., 1992). Since then, fMRI has broadened our understanding of how the brain functions under both healthy and diseased conditions (e.g., Rosen et al., 1998; Dolan, 2008; Haller and Bartsch, 2009; Rosen and Savoy, 2012). Although fMRI continues to grow in popularity in both research and clinical settings, the full potential of this technique remains untapped because fMRI activity has historically not been considered to be detectable in white matter tissue (Logothetis and Wandell, 2004). In spite of this, fMRI studies often produce activation in white matter and consequently there has been much debate over whether this activation is a true or false representation of underlying neural activity. There are two main reasons that white matter fMRI is controversial. First, BOLD signal relies on cerebral blood volume and flow, which are three to seven times lower in white matter (Rostrup et al., 2000; Preibisch and Haase, 2001; Helenius et al., 2003). However, the vasculature and perfusion of white matter (Figure 1) are capable of supporting hemodynamic changes that are detectable with BOLD fMRI [see Section White Matter Vasculature, Cerebral Blood Flow (CBF), and Cerebral Blood Volume (CBV)]. Second, the primary source of fMRI signal is thought to arise from post-synaptic potentials (which occur mainly in gray matter) as opposed to action potentials (e.g., Logothetis et al., 2001; but see e.g., Smith et al., 2002). However, neither of these statements exclude the possibility, and there is no direct evidence against the possibility of measuring fMRI activation in white matter.

White matter contains the connections between specialized processing regions and comprises approximately 50% of the human brain (Black, 2007; Arai and Lo, 2009; Harris and Attwell, 2012). The functional significance of white matter has been established by extensive lesion and anatomic studies, which have demonstrated the importance of intact white matter for normal brain function, and have implicated white matter damage and disconnections in numerous neurologic and psychiatric diseases (e.g., Catani and ffytche, 2005). A tool to non-invasively investigate the functional dynamics of white matter would substantially broaden current approaches to the study of brain connectivity, and may provide considerable insight into white matter diseases, such as multiple sclerosis.

## Previous reports of white matter fMRI activation

### BOLD fMRI

The majority of reports of BOLD fMRI activation in white matter involve the corpus callosum (Supplementary Table 1). Tettamanti et al. (2002) published a key report of white matter fMRI activation, in which a cluster was observed in the genu of the corpus callosum associated with a visuo-motor interhemispheric transfer task, the Poffenberger paradigm. In the Poffenberger task, light flashes are presented briefly (approximately 100 ms) to the right or left visual hemifield, and participants must respond with either the contralateral (crossed) or ipsilateral (uncrossed) hand (Poffenberger, 1912). Tettamanti et al. (2002) findings were confirmed by Omura et al. (2004), Weber et al. (2005), and Gawryluk et al. (2009). The involvement of the anterior corpus callosum in the Poffenberger paradigm is the most well-established example of white matter fMRI activation.

**Figure 2:**
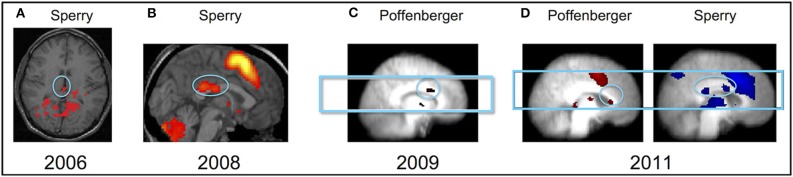
**Examples of white matter fMRI activation from studies published in 2006-2011. (A)** An exploratory study of white matter activation by D'Arcy et al. in 2006 used a Sperry interhemispheric transfer task and detected activation near the splenium of the corpus callosum. This initial study led to **(B)** a prospective study of fMRI activation in white matter, which used a similar task and also revealed activation in the posterior corpus callosum. A follow up study, **(C)** aimed to improve sensitivity to the detection of white matter fMRI activation, used a Poffenberger interhemispheric transfer task and detected a cluster of activation in the anterior corpus callosum. Taken together, this series of studies led to **(D)**, an investigation of whether different tasks could be used to functionally map different regions of the corpus callosum in the same group of individuals. The results were consistent with previous work and showed activation in the anterior corpus callosum during the Poffenberger task and posterior corpus callosum activation during the Sperry task.

Other interhemispheric transfer tasks have been used to target posterior regions of the corpus callosum. One such task is referred to as the Sperry paradigm, in recognition of the seminal work by Sperry and colleagues in which the function of the corpus callosum was probed by studying on split-brain patients (e.g., Gazzaniga et al., 1965; Myers and Sperry, 1985). In the Sperry task, word stimuli (for which the left hemisphere is relatively specialized) and face stimuli (for which the right hemisphere is relatively specialized) are presented briefly to the right or left visual hemifield. Crossed conditions involve presenting stimuli to the contralateral hemisphere (e.g., presenting word stimuli to the right hemisphere via the left visual field). D'Arcy et al. (2006) employed a Sperry task and an exploratory data analysis approach. Activation in the splenium of the corpus callosum activation could be observed, providing the first fMRI evidence of posterior callosal activation associated with an interhemispheric transfer task. Mazerolle et al. (2008) expanded and refined the approach taken by D'Arcy et al. (2006) by employing whole brain coverage, acquiring data at high field (4 T), and using a general linear model-based analysis technique. Splenium activation was observed in approximately 21% of individuals, as well as at the group level when liberal thresholds were applied (*p* < 0.005 uncorrected; Figure 2). Gawryluk et al. (2011a) went on to functionally map the corpus callosum within subjects, showing distinct areas of callosal activation for the Poffenberger and Sperry interhemispheric transfer tasks (Figure 2).

Recently, Fabri et al. (2011) noted that “a rising number of researchers have been reporting fMRI activation in white matter, particularly the corpus callosum.” Consequently, Fabri et al. (2011) sought to create a topographical map of the corpus callosum using data acquired from healthy participants at 1.5 T who completed tactile, gustatory, visual, and motor tasks. The results indicated that the corpus callosum was activated anteriorly by taste, at the midpoint by motor tasks, at the midpoint and posteriorly by tactile tasks, and posteriorly by visual stimuli. The central motor activation and posterior visual activation is consistent with previous findings from interhemispheric transfer tasks (Gawryluk et al., 2011a).

In addition to tasks aimed at eliciting interhemispheric transfer, tasks for investigating interhemispheric interactions have also been associated with callosal activation. Brandt et al. (2000) reported negative signal change in the occipital white matter containing the optic radiations contralateral to the visually stimulated hemisphere (uncorrected *p* < 0.001). However, this is the only report of task-related negative signal changes in white matter, making it difficult to interpret. Aramaki et al. (2006) reported corpus callosum activation associated with interhemispheric interactions during bimanual coordination.

Other conditions under which corpus callosum activation has been observed include a study of native signers viewing American Sign Language sentences with inflectional morphology (genu of the corpus callosum; Newman et al., 2010). Wang et al. (2013) used fMRI to study the neural correlates of acupuncture and detected activation in the posterior corpus callosum.

Outside of the corpus callosum, BOLD fMRI activation has been reported in the internal capsule. Mosier and colleagues reported activation associated with swallowing in the internal capsule, as well as the corpus callosum (Mosier et al., 1999a). Gawryluk et al. (2011b) found that activation could be detected in the internal capsule during a motor task (finger tapping), which was later confirmed by Mazerolle et al. (2013). In addition, white matter fMRI activation was observed for both healthy controls and Alzheimer's patients during a memory task (various white matter regions; Weis et al., 2011). Thyreau et al. (2012) also found a large number of white matter voxels above threshold in an investigation of random effects group statistics using a large database sample (*N* > 1500).

The studies reviewed above employed a task-based approach to investigate activation; that is, activation associated with the task/stimuli was evaluated. Activation associated with reaction time has also been explored in the context of white matter. In a multi-study analysis, Yarkoni et al. (2009) reported fMRI signal changes associated with reaction time in the right lateral genu of the corpus callosum, as well as in parts of the posterior corona radiata bilaterally (among gray matter regions). Shuster et al. (2014) used a similar reaction time based analysis of fMRI data related to speech production and detected activation in the mid-to-posterior corpus callosum.

Functional connectivity has also been observed in white matter during rest (i.e., task-free BOLD fMRI). Ding et al. (2013) examined temporal correlations of BOLD sensitive signals associated with resting state fMRI. Specifically, resting state fMRI data were used to evaluate the strength of functional connectivity within white matter tracts, which were defined using DTI tractography. Time courses from seed regions in the corpus callosum and optic radiations were considered. The results showed that functional connectivity was significantly greater for voxels within the same white matter tract than for random voxels (matched for distance from the seed). Thus, the correlation patterns associated with the seed regions were anisotropic: greater correlations were observed along white matter tracts than in other directions.

A small body of work exists that has attempted to better understand the factors related to sensitivity to BOLD fMRI activation in white matter. In an effort to increase sensitivity to the detection of fMRI signal in white matter, Gawryluk et al. (2009) investigated the underlying physics of white matter fMRI activation. The results showed that sensitivity to white matter clusters could be improved when T2-weighting was increased. However, this effect appears to be due to a general sensitivity increase from combining T2^*^- and T2-weighting, rather than a white matter specific effect (McWhinney et al., 2012). In another effort to increase sensitivity to the detection of white matter fMRI signal, Fraser et al. (2012) compared hemodynamic response functions (HRFs) in white and gray matter, and found similar response characteristics with reduced amplitude in white matter. Mazerolle et al. (2013) showed that field strength increases sensitivity to white matter fMRI activation, just as had been previously shown for gray matter. These studies have made progress toward understanding sensitivity to white matter activation; however, improving sensitivity to white matter activation remains an active area of research (discussed further in Sections Sensitivity and Artifacts and Basic Research).

**Figure 3:**
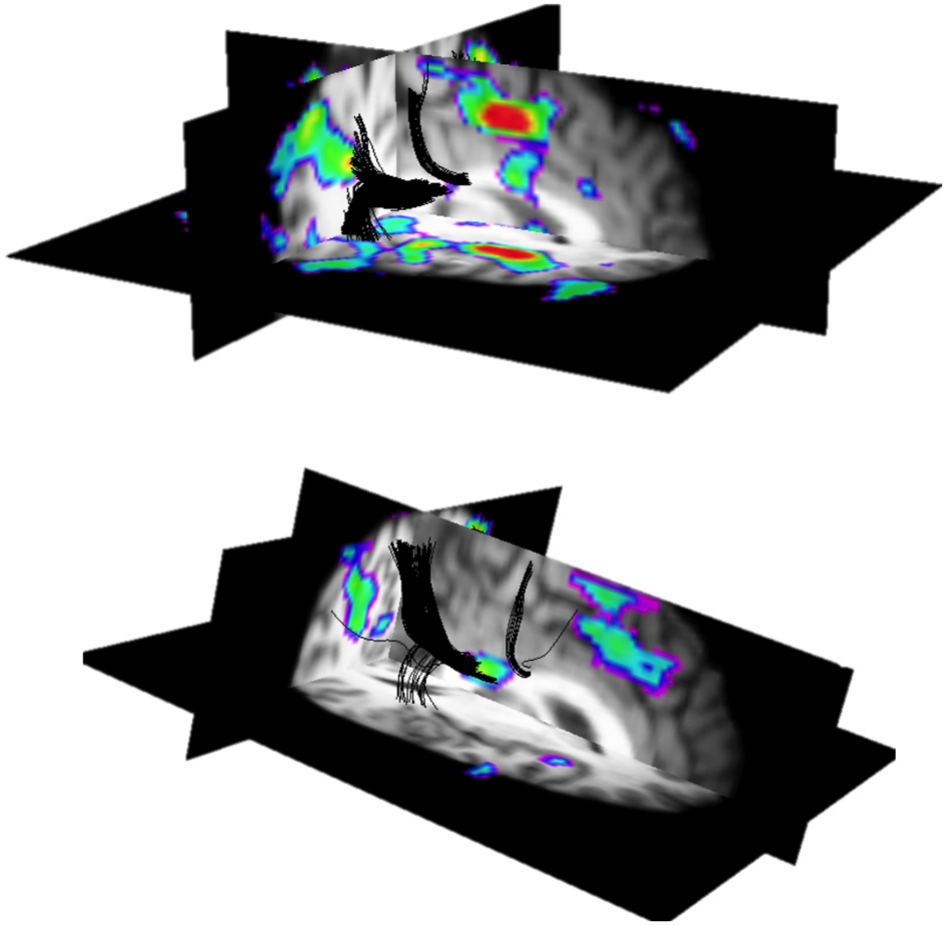
**3D views of white matter fMRI activation co-localized to functionally-guided tractography in two subjects**. An interhemispheric transfer task was used to elicit gray and white matter activation (rainbow color scale). Tracts (black) were seeded from regions of gray matter activation to determine whether the white matter activation was structurally connected to activation in gray matter. This work provided evidence for the anatomic basis of white matter activation; that is, regions of white matter activation are structurally connected to the activated network in gray matter. A discussion of individual variability in the location of callosal activation can be found in (Mazerolle et al., 2010). Figures were generated from data selected from (Mazerolle et al., 2010).

In order to better understand the relationship between white matter activation and the activated network in gray matter, functionally-guided tractography has been applied. Mazerolle et al. (2010) used tractography to verify that regions of callosal activation were structurally connected to cortical regions activated by an interhemispheric transfer task (Figure 3). Thus, there is an anatomic basis for the notion that white matter fMRI activation corresponds with activated distributed brain networks. These findings also highlighted the potential value of combining white matter fMRI activation and diffusion tensor imaging (DTI) tractography methods for brain connectivity research, an approach subsequently adopted by Fabri and Polonara (2013) for an expanded set of functional domains.

Supplementary Table 1 summarizes the methods and results of the studies in which white matter activation has been reported using BOLD fMRI. It is perhaps worth noting that the above list of studies reporting white matter fMRI activation may not be complete. It is difficult to identify all such studies on the basis of literature search, as white matter activation is not always reported in the title, abstract, or keywords. Nonetheless, in total, we have described 27 published reports of white matter BOLD fMRI activation. Compared to the explosion of BOLD fMRI publications over the past 20 years (Bandettini, 2012; Rosen and Savoy, 2012), the number of publications involving white matter fMRI activation remains relatively scarce. Given that there is a 50:50 ratio of white to gray matter tissue (Black, 2007), there is a notable discrepancy in the number of reports of white matter fMRI activation relative to the large number of publications of in the field as a whole. In Section Understanding why reports of white matter fMRI activation are uncommon, we describe the challenges that may explain the paucity of observations of white matter fMRI activation, including technical, physiological, and philosophical issues.

Taken together, the experiments that are readily identifiable provide strong support for the growing interest in this possibility and have paved the way for future research into the functional contribution of white matter. In the immediate term, the evidence to-date raises questions regarding the underlying characteristics of the fMRI signal, and its relationship to white matter tissue.

### Diffusion fMRI

In addition to the BOLD fMRI studies of white matter activation outlined in Section BOLD fMRI, there is emerging evidence that diffusion contrast might be sensitive to activity-dependent changes in white matter. Diffusion as a functional contrast mechanism has been reported for gray matter (e.g., Darquié et al., 2001; Williams et al., 2013). Task-correlated diffusivity changes are thought to represent structural changes induced by neural-activity-related cell swelling (Le Bihan, 2012). Recently, diffusion fMRI has been used to detect activation in white matter, specifically in the mouse optic nerve (Spees et al., 2013; Lin et al., 2014). Changes in white matter fractional anisotropy (FA), a tissue microstructure metric derived from DTI, have also been reported for a visual task; however, FA must be averaged across large tracts of interest in order to observe this effect (Mandl et al., 2008). While more work is needed to (1) understand the physiological bases of functional contrast measured using diffusion, (2) improve the sensitivity of diffusion fMRI to activation, and (3) understand the impact of tissue type on functional diffusion contrast, diffusion fMRI remains an exciting alternative approach to understand the functional dynamics of the brain. Because diffusion fMRI contrast is not thought to rely on hemodynamic changes, it has potential to confer significant advantages for detecting white matter activation over BOLD fMRI (but see Miller et al., 2007; Ding et al., 2012 for evidence of hemodynamic contributions to the diffusion fMRI signal).

## The possible physiological bases of BOLD fMRI signal in white matter

### White matter vasculature, cerebral blood flow (CBF), and cerebral blood volume (CBV)

The vasculature in white matter has been described based on histological methods (Lierse and Horstmann, 1965; Duvernoy et al., 1981), as well as more recently with novel MRI methods. These studies have estimated that the venous vessels in white matter are, on average, approximately the same size as those in gray matter, although the white matter venous vessel density is approximately half the value of gray matter (Jochimsen et al., 2010; Table 1). The microvasculature density of white matter has been estimated using MRI techniques to range from 10–192 microvessels/mm^2^ for white matter versus 99–761 microvessels/mm^2^ for gray matter (Jensen et al., 2006). Reduced vascular density can also be observed histologically (Figure 1A; Lierse and Horstmann, 1965). In addition, blood vessels in white matter can be clearly seen during neurosurgery. For example, Figure 1B depicts a blood vessel on the surface of the corpus callosum.

**Table 1:** **Factors that influence the ability to detect fMRI activation and how they differ across tissue types**.

	**Gray matter**	**White matter**	**Predicted effect on fMRI activation**
Cerebral blood flow (CBF)	50–100 ml/100 g/min (Rostrup et al., 2000; Preibisch and Haase, 2001; Helenius et al., 2003)	10–30 ml/100 g/min (Rostrup et al., 2000; Helenius et al., 2003; Preibisch and Haase, 2001)	Reduced maximal amplitude of fMRI responses in white matter.
Cerebral blood volume (CBV)	4.6 ml/100 g (Helenius et al., 2003)	1.3 ml/100 g (Helenius et al., 2003)	Reduced maximal amplitude of fMRI responses in white matter.
	1.0–3.3% capillaries by volume (Lierse and Horstmann, 1965)	0.3–0.9% capillaries by volume (Lierse and Horstmann, 1965)	
Venous vessel size	13.4 micron radius (Jochimsen et al., 2010)	13.7 micron radius (Jochimsen et al., 2010)	If the vessels in white matter are of equal or greater size than those in gray matter, one might expect a tendency toward greater sensitivity in white matter for standard gradient-echo BOLD sequences (Boxerman et al., 1995). However, it is likely that the effect of lower CBF and CBV in white matter is the dominant factor, consistent with the notion of reduced sensitivity to activation in white matter.
	10–63 micron radius (intracortical veins; Duvernoy et al., 1981)	30–60 micron radius (Duvernoy et al., 1981)	
T2^*^	89.3 ms (1.5 T)	71.7 ms (1.5 T)	Optimal TE depends on T2^*^; standard fMRI parameters may not be optimized for detecting white matter activation, particularly at 1.5 T due to greater differences between the tissue types.
	59.7 ms (3 T)	54.6 ms (3 T)	
	N.B. Values for cortical gray matter (Peters et al., 2006)	(Peters et al., 2006)	
Physiological noise	Higher (Bodurka et al., 2007)	Lower (Bodurka et al., 2007)	White matter fMRI signals may be less contaminated by physiological noise than gray matter.
Tissue geometry	Cortical gray matter has substantial potential for PVE with CSF in sulci	Some areas of white matter are very uniform; others neighbor gray matter and/or the lateral ventricles	PVEs are problematic throughout white and gray matter.
Categories of neural activity	Post-synaptic potentials and action potentials	Mostly action potentials	Both are linked to BOLD fMRI signal changes (e.g., Logothetis et al., 2001; Smith et al., 2002).
Presence of activity-dependent metabolic changes	Observed using numerous techniques, including calibrated fMRI (Hoge et al., 1999), PET (Magistretti, 2006), and optical imaging (Devor et al., 2012)	Observed using autoradiology (Weber et al., 2002)	While white matter supports activity-dependent metabolic changes, the autoradiography evidence does not provide sufficient temporal resolution to imply that such changes might be detectable with BOLD fMRI.
Astrocytes	Positioned to facilitate neurovascular coupling (Petzold and Murthy, 2011)	Positioned to facilitate neurovascular coupling (Petzold and Murthy, 2011)	Underlying neurophysiology of gray and white matter fMRI activation may share overlapping components.

Given the lower density of vessels, it follows that white matter has lower CBF and CBV relative to gray matter (Rostrup et al., 2000; Preibisch and Haase, 2001; Helenius et al., 2003; Table 1). Recent advances in perfusion MRI imaging with arterial spin labeling are allowing white matter cerebral blood flow to be detected in normal healthy volunteers (Figure 1C; Van Osch et al., 2009; Gardener and Jezzard, 2014), providing further confirmation that white matter blood flow is one-tenth to one-third that of gray matter (Table 1). For this reason, it was previously thought that fMRI was sensitive exclusively to gray matter (Logothetis and Wandell, 2004). However, hemodynamic changes can be detected in white matter during vascular challenges, such as breath-holding tasks or hypercapnia (Rostrup et al., 2000; Preibisch and Haase, 2001; Helenius et al., 2003; Macey et al., 2003; Van der Zande et al., 2005; Mandell et al., 2008; Driver et al., 2010; Thomas et al., 2014). Thus, there is evidence that white matter has the vascular capacity to support hemodynamic changes that are detectable with fMRI, particularly at higher magnetic field strengths.

### Neurovascular and neurometabolic coupling in white matter

A crucial question that remains, however, is whether activity-dependent hemodynamic changes take place in white matter. Activity-dependent hemodynamic changes are a necessary feature for any neural activity to be detectable with fMRI. Investigations into the neurophysiologic source of fMRI signals have linked the signal to post-synaptic potentials, which are mainly localized to gray matter. For example, Logothetis' group showed that BOLD signal changes require only local field potentials (LFPs, which reflect post-synaptic potentials) and can take place in the absence of spiking activity (Rauch et al., 2008). Although there are some synapses and even some cell bodies in white matter (Kukley et al., 2007; García-Marín et al., 2010), post-synaptic potentials account for less than 1% of the energy demands in white matter (Harris and Attwell, 2012), making white matter post-synaptic potentials unlikely to account for the majority of white matter fMRI signal changes. In white matter, the primary type of neural activity is spiking (which reflects action potentials). Although there is evidence provided that LFPs are sufficient for BOLD signal changes (e.g., Rauch et al., 2008), a relationship between spiking and fMRI activation has also been reported (e.g., Smith et al., 2002), and it remains possible that spiking activity is sufficient for inducing a hemodynamic response (Table 1). It is important to note that the relationship between neural activity and hemodynamic changes has only been studied for gray matter, where post-synaptic potentials do require the majority of energy (Attwell and Laughlin, 2001; Harris and Attwell, 2012).

Given that spiking and LFP activity is inherently correlated, and neurovascular coupling of spikes in the absence of resulting LFPs has not be studied, it is difficult to draw conclusions regarding whether spikes elicit hemodynamic responses. Thus, we consider whether the energetic demands of spiking activity are consistent with a requirement for a measurable hemodynamic response. Spiking is indeed associated with increased activity at energy-dependent ion pumps (Erecińska and Dagani, 1990; Aiello and Bach-y-Rita, 2000). The brain does not have any major energy or oxygen reserves, relying on blood perfusion to meet its metabolic demands (Lecrux and Hamel, 2011). Thus, any spiking related energy demands might result in a hemodynamic response. A crucial factor that remains is whether the magnitude of activity dependent metabolic demands in white matter might reasonably be expected to produce changes of sufficient magnitude to be detected with fMRI.

Harris and Attwell (2012) have proposed an energy budget for white matter, in which they estimate that the energy associated with spiking in white matter comprises only 0.4–7% of the total energy demand in white matter (depending on myelination status). The vast majority of white matter energy demands are thought to arise from maintaining the resting potential (independent of restoring the ionic gradients due to action potentials) and housekeeping processes such as macromolecule turnover and axoplasmic transport.

A feature not included in Harris and Attwell's (2012) energy budget for spiking is the potential involvement of non-neuronal cell types. Astrocytes exist in white matter (Waxman and Ritchie, 1993; Rash, 2010; Harris and Attwell, 2012), and may perform similar functional roles as gray matter astrocytes. One example is K^+^ siphoning, in which the increase in extracellular K^+^ concentration associated with spiking activity results in increased K^+^ uptake into astrocytes. Kalsi et al. (2004) found that astrocyte expression of K^+^ channels is localized to perivascular end feet and on processes located within myelinated axon bundles, consistent with a K^+^ siphon function. It has been proposed that K^+^ is first transported across layers of myelin via gap junctions before being taken up by astrocytes (Rash, 2010). Astrocytes then release K^+^ perivascularly, which relaxes the smooth muscle cells of the arterioles surrounded by the astrocytes' end feet. Thus, K^+^ siphoning is associated with hemodynamic changes, and has been speculated to contribute to neurovascular coupling in gray matter (Petzold and Murthy, 2011). The presence of K^+^ channels in the astrocytes and oligodendrocytes of white matter have been confirmed for the rat optic nerve (Rash, 2010). While diffusion of K^+^ through gap junctions is not an energy-demanding process, as it is driven by an electrochemical gradient, this may be an important pathway linking action potentials and a hemodynamic response.

Recently, Barbaresi et al. (2013) investigated the presence and distribution of nitric oxide (NO) producing neurons in the corpus callosum. Immunohistochemistry techniques revealed NO producing neurons throughout the corpus callosum, with higher density in lateral and caudal regions. Notably, the somatic, dendritic, and axonal processes of many NO producing neurons were found nearby blood vessels in the corpus callosum. Given that NO is a powerful vasodilator, the authors concluded that “NO positive neurons transduce neuronal signals into vascular responses in selected [callosal] regions, thus giving rise to hemodynamic changes detectable by neuroimaging.”

In addition to the neurovascular mechanisms speculated above, experimental evidence of activity dependent metabolic changes in white matter has been established. Weber et al. (2002) investigated activity-dependent glucose uptake changes in a rodent model using [18F]fluorodeoxyglucose autoradiography. They reported increased glucose uptake in the corpus callosum associated with electrical stimulation of a cortical region with callosal projections. Furthermore, the increase in glucose uptake was greater for higher frequency stimulation. However, it has not yet been experimentally demonstrated that activity-dependent increases in glucose uptake in white matter are coupled to a hemodynamic response that could be measured with fMRI. In addition, the lack of temporal information available from autoradiography is problematic; the observed increased in metabolic demands associated with spiking rate may occur over too slow a time scale (i.e., many minutes or hours) for this effect to be relevant for typical fMRI experiments (Table 1). For these reasons, it will be important for future studies to experimentally verify whether such metabolic increases are accompanied by fMRI signal changes.

## Understanding why reports of white matter fMRI activation are uncommon

As previously stated by Tettamanti et al. (2002), Yarkoni et al. (2009), and Mazerolle et al. (2008), the primary argument against white matter fMRI activation is the historical lack of reports, rather than any fundamental property that would preclude its existence. In fact, it has recently been speculated that fractional changes in fMRI signals may be greater when baseline flow is lower such as in white matter (Ding et al., 2013). In this section, we put forward some explanations for the paucity of white matter fMRI reports and offer rationales to change the common practice of dismissing fMRI signal in white matter.

### Sensitivity and artifacts

#### Acquisition

One reason that fMRI activation in white matter remains scarce in the literature is that signals associated with fMRI activation in white matter are lower in magnitude than those associated with gray matter activation. The smaller signal changes in white matter may be difficult to detect at conventional field strengths (1.5 T). In fact, there is direct evidence that sensitivity to white matter activation increases with field strength (Mazerolle et al., 2013), a phenomenon, which has been extensively reported in gray matter (e.g., Gati et al., 1997). Reports of white matter activation may also be relatively limited because fMRI acquisition parameters have been optimized for detection of gray matter activation. For example, T2 and T2^*^ are different between white and gray matter (Peters et al., 2006; see Table 1), such that the optimal TE may differ between the tissue types.

#### Analysis

In addition to acquisition parameters being tailored for gray matter fMRI activation, it is also important to consider potential biases introduced by analysis techniques. Because fMRI is an indirect measure of functional brain activation, the validity of results is regularly evaluated based on what is already known about the functional organization of the brain. That is, because there is rarely a gold standard available for fMRI results to be compared against, experimenter expectations tend to play a major role in evaluating data quality and the validity of results. This sentiment was described by Poline et al. (2006): “neuroscientists or clinicians [typically] assess [fMRI] results in relation to their prior expectations, although this biases fMRI studies away from making new and unexpected discoveries” (p. 352).

For the vast majority of fMRI analyses, the shape of the HRF is assumed. However, even within gray matter, there is evidence that the shape of the HRF varies between regions (Miezin et al., 2000; Handwerker et al., 2004; Gonzalez-Castillo et al., 2012). Research into the shape of the HRF in white matter has produced conflicting results. One study showed that the shape of the HRF in white matter is the same as the canonical HRF. However, as an initial step, this study restricted the analysis to regions in which activation could be detected using the canonical HRF (Fraser et al., 2012). Other work has demonstrated slower responses in white matter compared to gray matter (Yarkoni et al., 2009). In the case that differences between the gray matter and white matter HRF exist, the use of the gray matter canonical HRF for modeling activation could result in systematic underestimation of white matter activation. More research is needed to evaluate differences in the shape of the HRF between gray matter and white matter, as well as among different white matter regions. Then, the potential benefits of regionally specific HRFs for detecting white matter fMRI activation could be evaluated.

One major analysis practice, which directly biases against detecting white matter fMRI activation is the use of white matter signals as a nuisance regressor (e.g., Leber, 2010). This approach has been recommended as best practice for resting state functional connectivity analyses (Van Dijk et al., 2010). White matter signals have also been used to estimate physiological noise in fMRI data (e.g., Behzadi et al., 2007). In addition, white matter signals have been used to set thresholds such that, for activation to be considered significant, its intensity must exceed that of white matter (Soltanian-Zadeh et al., 2004). Decreases in white matter fMRI activation have also been cited as evidence that a novel denoising procedure was successful (Tohka et al., 2008). This approach is sensible if and only if there are no signals of interest in white matter. If we assume that the existence of interesting signals in white matter cannot be ruled out, then the validity of assigning white matter signals to the noise pool rests on whether white matter is more prone to artifacts than gray matter. For example, Weis et al. (2011) states: “standard EPI sequences as used [by Weis et al. (2011)] are not optimal for detecting white matter activations and the risk of artifacts is augmented” (p. 388). This statement raised the question: what could cause an increase in artifactual activations in a manner specific to white matter, such that selectively discounting signals in white matter would be a valid approach?

#### Artifacts

Below, we outline common sources of artifact and consider whether they might preferentially contaminate white matter.

***Motion***. A common cause of artifactual activation is task-related motion. When participant motion is task-correlated, artifactual activations can occur, particularly at tissue boundaries (Johnstone et al., 2006). Thus, voxels near the boundaries between gray matter and white matter, and between brain parenchyma and CSF, will be most sensitive to motion-induced artifactual activations. White matter voxels *per se* are unlikely to be more susceptible to false activations due to motion than other voxels. Regions of deep white matter, which can be centimeters away from tissue boundaries, may be particularly insensitive to such artifacts. This is because, in deep white matter, it is unlikely that tissue boundaries would be displaced, which causes apparent changes in signal intensity that can be misidentified as activation. Furthermore, many studies of white matter activation have mitigated the risk of motion-related activations by including the estimated motion parameters (output by the motion correction) as regressors of no interest in the analysis model (Mazerolle et al., 2008; Yarkoni et al., 2009; Gawryluk et al., 2011a,b). Even with this approach, it is possible that nonlinear spin history effects caused by motion are not corrected (Yancey et al., 2011). However, spin history effects are likely to be most severe near regions of large magnetic gradients (i.e., susceptibility induced field gradients near the sinuses) and would not be expected to result in white matter-specific effects.

***Partial volume effects***. Another reason that some groups have questioned fMRI activation in white matter is the possible attribution of the signal to partial volume effects. Partial volume effects can occur when a voxel exists at the interface multiple tissue types, which renders the source of the signal indistinguishable. This problem can be exacerbated by the use of low-resolution (large) voxels (which offer benefits in SNR) as well as data pre-processing steps such as spatial smoothing. It is possible, for example, that fMRI activation in the internal capsule (Mosier et al., 1999a; Gawryluk et al., 2011b) is the result of signal contamination from nearby subcortical gray matter structures (e.g., Mortiz et al., 2000; Lehéricy et al., 2006). In fact, partial volume effects are a universal concern in fMRI; however, there are a few key pieces of evidence that the activation in white matter is not a result of partial volume effects from gray matter signal.

First, the areas in which white matter activation have been reported are functionally consistent with the nature of the given task. For example, activation in the corpus callosum was observed during interhemispheric transfer tasks, as would be expected (Gawryluk et al., 2011a). Second, in the case that a cluster was large enough to cover both white and gray matter voxels, many studies have ensured that a local maximum was co-localized to the white matter structure of interest (e.g., Mazerolle et al., 2013). Indeed, many studies have reported activation that is entirely bounded by white matter tissue, with no adjacent gray matter (e.g., Fraser et al., 2012). Recent work by Mazerolle (2012) has provided further evidence that white matter activation is not a result of partial volume effects. This work also took an analysis approach that used very conservative masks of gray and white matter (thereby reducing/eliminating partial volume effects) on a data set that showed whole brain activation (from a breath hold task). It was determined that the activation seen in white matter was could not be explained solely on the basis of gray matter partial volume (Mazerolle, 2012). Therefore, although partial volume effects should always be considered, it is unlikely that the majority of white matter activation reported can be attributed to partial volume effects (Table 1).

***Physiological noise***. Another potential source of artifactual activation is physiological noise. When task-correlated, respiratory and cardiac artifacts can result in artifactual activation. Task-correlated respiratory and/or cardiac rate changes have been observed for tasks that require attention, are cognitively challenging, or are emotional (Birn et al., 2009). However, Birn et al. (2006) demonstrated that respiratory related artifacts tend to be most problematic in gray matter or near large vessels. This is consistent with findings that tSNR is greater in white matter compared to gray matter (e.g., Bodurka et al., 2007; Gonzalez-Castillo et al., 2011; Mazerolle et al., 2013). Thus, it is unlikely that white matter is particularly prone to artifactual activation caused by physiological noise (Table 1). Thus, it may be worthwhile re-considering current approaches that define noise to be white matter signals (see Section Analysis), especially given that other voxels in which no neural tissue is contained are available (e.g., CSF).

## Future directions in white matter fMRI research

### Basic research

There is a growing interest in using brain imaging techniques to describe the entire network of connections in the human brain (i.e., map the human connectome; e.g., Sporns, 2012). This research is critical, given that intact brain connectivity is necessary for normal function, and disconnections are implicated in many neurologic and psychiatric conditions (e.g., Charil et al., 2003; White et al., 2008). However, the current methods of studying connectivity lack a direct approach. To date, MRI studies of connectivity have mainly used DTI tractography and functional connectivity analyses. While DTI tractography has permitted significant advances in the current understanding of brain connectivity, the technique is limited to evaluating structure, and does not provide information about the functional dynamics of the identified networks. Information from DTI tractography can be augmented with functional connectivity analyses, which typically infers connectivity based on correlations between the time series of different gray matter regions. Even when combined, DTI tractography and functional connectivity approaches are fundamentally limited by the fact that neither evaluates the functional dynamics directly within white matter pathways. Studying the activation patterns of the white matter pathways is crucial for understanding the interactions among different nodes in a brain network, as well as evaluating whether structural white matter changes are associated with functional changes. This approach may also clarify whether observed correlations between gray matter result from direct connections, or indirect connections mediated by other gray matter areas.

Basic research could also focus on analysis techniques to further optimize the sensitivity to the detection of white matter fMRI activation. For example, it may be possible to increase sensitivity to white matter activation by using a white matter specific HRF. Although there is evidence that the HRF in the corpus callosum resembles the canonical HRF (Fraser et al., 2012), more work is needed to evaluate the HRF in various white matter regions. Relatedly, various fMRI analysis software packages handle data differently (e.g., motion correction, thresholding options; Oakes et al., 2005). Future work could compare different analysis options and determine the selections that are most sensitive to activation in white matter.

A key step to move the field forward will be investigating the neurophysiological basis of white matter fMRI activation. The neural events and signaling pathways underlying fMRI signals are still not fully understood. The vast majority of work on neurovascular coupling has focused on gray matter. By applying this methodology to white matter, we could better understand the underpinnings of white matter fMRI activation. This knowledge would both improve confidence in white matter fMRI activation and enhance the interpretations of such findings.

### Clinical research

The ability to detect fMRI activation in white matter has clear implications for the evaluation of white matter disease and damage. A key step in linking advances in research to the evaluation of clinical disorders is to use clinical measures of white matter function in fMRI studies. Recently, Gawryluk and colleagues adapted the Symbol Digit Modalities Test, a sensitive measure of processing speed that is associated with white matter integrity (Chiaravalloti and Deluca, 2008) for use with fMRI. The results revealed activation in the corpus callosum and/or internal capsule in 88% of participants (*N* = 17; Gawryluk et al., 2014). The next step in this line of research is to study white mater activation in patient groups. This will be particularly relevant given that findings may vary by disease or condition of the patient and may provide insight into discrepancies between structural neuroimaging findings and symptomology (Pelletier et al., 2009).

Another consideration in terms of preparing white matter fMRI for clinical applications, the main challenge will be in developing a comprehensive approach. The majority of the white matter fMRI studies to date have elicited activation in the corpus callosum and internal capsule. Although these structures are often involved in white matter disorder, an ideal and thorough assessment of white matter function should be able to evaluate multiple regions. Such an evaluation would likely require a battery approach in which a variety of short tasks could be administered to study specified areas of interest. Tests developed for a battery should also be studied in a large group of individuals to document the variability associated with a given task.

However, a battery approach may not always be practical given constraints on scanner time and patient comfort and performance. In gray matter, the suitability of a task-free (i.e., resting state) connectivity approach to evaluate the functional status of the tissue in all brain networks has recently been considered, with promising results (e.g. Liu et al., 2009). This has significant advances over a task battery approach with respect to scan duration and patient compliance requirements. While there may be additional hurdles applying this approach to the functional assessment of white matter given the smaller magnitude of changes expected, recent results demonstrating resting state functional connectivity within white matter pathways is promising (Ding et al., 2013).

Whether a battery or task-free functional connectivity approach is used, quantification of within-subject reliability of white matter activation will be necessary to interpret results in patients. Reliability is an active area of fMRI research. Varying degrees of reliability have been reported for gray matter activation, depending on the analysis technique, reliability metric, test-retest interval, as well as the particular tasks and regions of interest (reviewed in Bennett and Miller, 2010). Regional differences in reliability may be explained in part by the local microvasculature. For example, McGonigle et al. (2000) speculated that high variability in visual cortex may be partially due to the high concentration of venules in the region, allowing a wider range of responses to visual stimulation. Thus, it is important to evaluate reliability specifically for white matter fMRI activation, particularly given the known differences in vasculature, CBF, and CBV in white matter relative to gray matter (Duvernoy et al., 1981; Rostrup et al., 2000; Preibisch and Haase, 2001; Helenius et al., 2003). Clinically, this question becomes highly relevant when assessing patients with white matter disorder over time in order to distinguish between real changes versus normal variability.

## Conclusions

Functional MRI is a tremendous technique that has allowed for noninvasive visualization of functional dynamics in the human brain. However, a major limitation of fMRI is that it is typically only used to study gray matter, which merely represents half of the brain. Conventionally, white matter tissue has only been examined using structural measures that do not always correlate well with measures of function (Pelletier et al., 2009). However, a growing body of work supports the notion that fMRI can be used to study functional dynamics in white matter. This approach is increasingly being applied to provide novel insights into brain connectivity. The ability to detect activation in the brain's connections will open new research avenues into how the regions within brain networks interact to support complex cognitive functions. White matter fMRI will also provide valuable insight into a number of disorders and conditions, including multiple sclerosis, diffuse axonal injury, degenerative diseases, and neurosurgical patients. Taken together, the advances presented in this review provide support for a more direct method of studying and evaluating white matter function with fMRI.

## Author contributions

Jodie R. Gawryluk and Erin L. Mazerolle generated the content and drafted the review manuscript. Ryan C. N. D'Arcy led the research program and revised the review manuscript draft.

### Conflict of interest statement

The authors declare that the research was conducted in the absence of any commercial or financial relationships that could be construed as a potential conflict of interest.
